# Integrated Metabolomics and Transcriptomics Analyses of the Biosynthesis of Arbutin and 6′-*O*-Caffeoylarbutin in *Vaccinium dunalianum* Cell Suspension Cultures Fed with Hydroquinone

**DOI:** 10.3390/ijms25147760

**Published:** 2024-07-16

**Authors:** Churan Li, Boxiao Wu, Weihua Wang, Xiaoqin Yang, Yun Liu, Guolei Zhu, Sida Xie, Qian Jiang, Yong Ding, Yingjun Zhang, Ping Zhao, Lihua Zou

**Affiliations:** 1 Key Laboratory of State Forestry and Grassland Administration on Highly-Efficient Utilization of Forestry Biomass Resources in Southwest China, Southwest Forestry University, Kunming 650224, China; churanli@swfu.edu.cn (C.L.); wbx1437@swfu.edu.cn (B.W.); 34016@ztu.edu.cn (W.W.); yangxiaoqin@swfu.edu.cn (X.Y.); guoleizhu@163.com (G.Z.); dream102035@163.com (S.X.); m17368708632@163.com (Q.J.); 2 Key Laboratory for Forest Resources Conservation and Utilization in the Southwest Mountains of China Ministry of Education, Southwest Forestry University, Kunming 650224, China; liuyun@swfu.edu.cn (Y.L.); dingyong@swfu.edu.cn (Y.D.); 3 State Key Laboratory of Phytochemistry and Plant Resources in West China, Kunming Institute of Botany, Chinese Academy of Sciences, Kunming 650201, China; zhangyj@mail.kib.ac.cn

**Keywords:** *Vaccinium dunalianum*, cell suspension culture, precursor feeding, arbutin, 6′-*O*-caffeoylarbutin, metabolomics, transcriptomics

## Abstract

Arbutin and 6′-*O*-caffeoylarbutin (CA) from *Vaccinium dunalianum* Wight are known for their ability to inhibit melanin synthesis. To boost the production of arbutin and CA, precursor feeding with hydroquinone (HQ) was studied in *V. dunalianum* suspension cells. The effect of HQ on the biosynthesis of arbutin and CA in the suspension cells was investigated using high-performance liquid chromatography (HPLC), and possible molecular mechanisms were analyzed using metabolomics and transcriptomics analyses. HPLC analysis only showed that the addition of HQ significantly enhanced arbutin synthesis in cells, peaking at 15.52 ± 0.28 mg·g^−1^ after 0.5 mmol·L^−1^ HQ treatment for 12 h. Subsequently, metabolomics identified 78 differential expression metabolites (DEMs), of which arbutin and CA were significantly up-regulated metabolites. Moreover, transcriptomics found a total of 10,628 differential expression genes (DEGs). The integrated transcriptomics and metabolomics revealed that HQ significantly enhanced the expression of two arbutin synthase (AS) genes (Unigene0063512 and Unigene0063513), boosting arbutin synthesis. Additionally, it is speculated that CA was generated from arbutin and 3,4,5-tricaffeoylquinic acid catalyzed by caffeoyl transferase, with Unigene0044545, Unigene0043539, and Unigene0017356 as potentially associated genes with CA synthesis. These findings indicate that the precursor feeding strategy offers a promising approach for the mass production of arbutin and CA in *V. dunalianum* suspension cells and provides new insights for CA biosynthesis in *V. dunalianum*.

## 1. Introduction

Arbutin is a hydroquinone glucoside compound found in various natural sources, such as bearberry, pears, mulberry, cranberry, wheat, coffee, and tea [1,2]. It exhibits beneficial properties of antioxidant, anti-inflammatory, antimicrobial, and anticancer [3,4,5]. Arbutin serves as a natural skin-lightening agent owing to its ability to inhibit tyrosinase to hinder melanin synthesis [6]. 6′-*O*-Caffeoylarbutin (CA) is an arbutin derivative that possesses better anti-melanin activity and lower toxicity than arbutin [7]. Several studies have suggested that CA had antioxidant and hepatoprotective effects, and showed potential as an anti-COVID-19 agent [8,9]. Therefore, it is of great significance to achieve large-scale production of arbutin and CA. Notably, *Vaccinium dunalianum* Wight is a rich source of CA and arbutin. *V. dunalianum* belongs to the genus *Vaccinium* of the family Ericaceae, which is a traditional herbal medicine with the activities of analgesia and promoting blood circulation [9]. Phytochemical analysis indicated that the leaf buds of *V. dunalianum* are rich in arbutin and CA, among which CA is the most abundant with a content of 31.76% [10,11]. The high accumulation of CA and arbutin in *V. dunalianum* and their favorable activity make it a valuable natural resource. However, the yield and purity of secondary metabolites obtained by the extraction method are low and are vulnerable to external conditions. Thus, alternative strategies are urgently required to meet the mass market demand for arbutin and CA while mitigating habitat destruction.

Plant tissue culture systems, especially cell suspension culture, are widely used for the mass cultivation of medicinal plants. This approach allows for the production of secondary metabolites while avoiding high production costs and the destruction of wild plant resources [12,13]. So far, cell suspension culture technology has been successfully applied to the production of various metabolites, including terpenoids [13], phenolic acids [14], and flavonoids [15,16]. However, achieving sufficient metabolite production from some plant cell suspension cultures remains challenging without proper induction conditions. Common strategies to enhance metabolite content in cell suspension cultures include optimization of culture conditions, elicitation, and precursor feeding. Among these methods, precursor feeding involves supplementing the required precursor to boost the production of target metabolites. Previous studies have attempted to promote the production of diverse metabolites from various plants through precursor feeding in cell suspension cultures. Luo and He demonstrated that paclitaxel production could be increased by supplementing precursors in *Taxus chinensis* (Pilg.) Rehder suspension cells [17]. Similarly, John et al. proved that the addition of cholesterol and hydrocholesterol increased the 20-hydroxyecdysone content in *Achyranthes aspera* Linn. cell suspension cultures [18]. Furthermore, Rattan et al. effectively transformed tyrosol into salidroside in *Rhodiola imbricata* Edgew. suspension cells [19].

Previously, cell suspension cultures of *V. dunalianum* were established with the aim of mass-producing arbutin and CA [20]. However, a low content of CA (0.44%) was detected in *V. dunalianum* suspension cells, whereas no arbutin was detected through liquid chromatography–mass spectrometry (LC-MS) analysis [21]. Recent studies have approved hydroquinone (HQ) as a precursor for arbutin synthesis in plant cell cultures [22]. Meanwhile, CA is a is a caffeoyl conjugate at the glucose-6′ position of arbutin, but the specific caffeoyl-transferase involved in its synthesis remains unknown [23]. Although the synthetic pathway of CA has not been clarified, it is speculated that enhancing arbutin synthesis may positively influence CA biosynthesis. Additionally, precursor feeding can promote the accumulation of plant secondary metabolites, resulting in changes in both metabolites and genes. Transcriptomics and metabolomics are powerful high-throughput technologies and invaluable in elucidating the synthesis pathways of important secondary metabolites in plants by identifying differentially expressed genes and metabolites [24,25,26]. In recent years, the combined analysis of transcriptomics and metabolomics has been widely used in studying the metabolic pathway of plant bioactive compounds [27,28].

Therefore, this study selected HQ as a precursor and investigated its effect on the accumulation of arbutin and CA in suspension cells of *V. dunalianum*. Furthermore, integrated metabolomics and transcriptomics were performed to analyze the molecular mechanism of the effects of HQ on the synthesis of arbutin and CA in suspension cells. This study aims to provide a promising biotechnological strategy for the production of arbutin and CA and give new insights into the biosynthetic mechanisms of arbutin and CA in the cell suspension cultures of *V. dunalianum*.

## 2. Results

### 2.1. Effect of HQ on Metabolites in V. dunalianum Suspension Cells by High-Performance Liquid Chromatography (HPLC)

**Figure 1:**
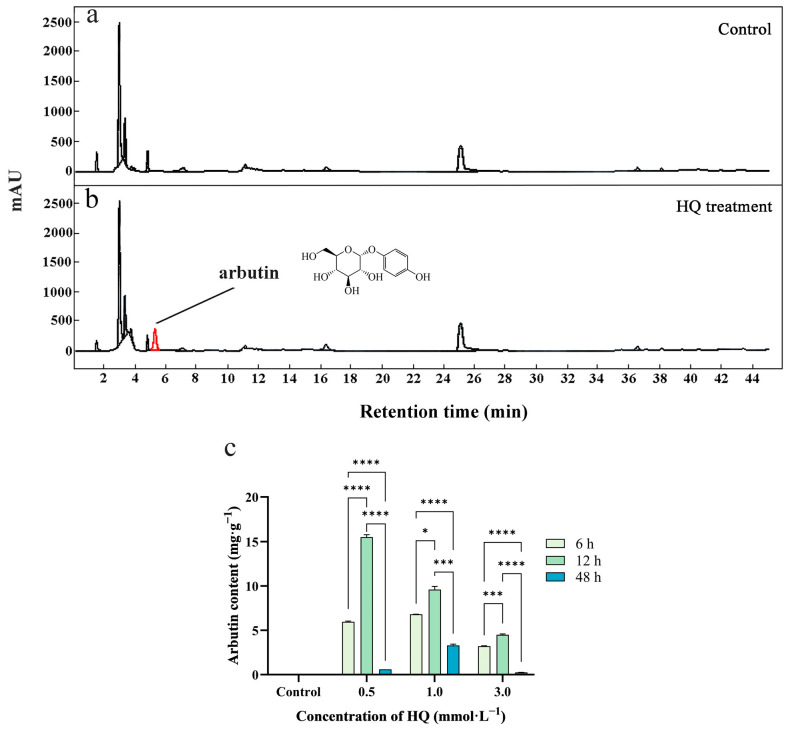
The effect of HQ on the biosynthesis of main compounds in *V. dunalianum* suspension cells. (**a**) HPLC chromatograph of the extracts suspension cells in the control group. (**b**) HPLC chromatograph of the extracts suspension cells in HQ treatment group. (**c**) Arbutin content in suspension cells treated with different concentrations of HQ at different times. The data was the means of three replicates (n = 3), and the vertical bars represented the standard error of replicates. Asterisk symbols (*) indicated significant differences according to Duncan’s multiple range test (* *p* < 0.05; *** *p* < 0.001; **** *p* < 0.0001).

To investigate the effect of precursor HQ addition on the metabolites of *V. dunalianum* suspension cells, methanol extracts from control and HQ-treated groups were analyzed using HPLC. In the control group, neither arbutin nor CA was detected in suspension cells during culture (Figure 1a). However, the presence of arbutin was observed in the suspension cells of the HQ-treated group (Figure 1b). As shown in Figure 1c and Table 1, the addition of HQ at varying concentrations (0.5, 1.0, and 3.0 mmol·L^−1^) significantly promoted the biosynthesis of arbutin. After 12 h of treatment, the maximum production of arbutin in the suspension cells was determined, which was 15.52 ± 0.28, 9.58 ± 0.38, and 4.49 ± 0.10 mg·g^−1^ under the treatments of 0.5, 1.0, and 3.0 mmol·L^−1^ HQ, respectively (Figure 1c and Table 1). Specifically, treatment with 0.5 mmol·L^−1^ HQ for 12 h was most effective in enhancing the synthesis of arbutin in suspension cells. Under the same concentration of HQ treatment, the arbutin content in suspension cells increased initially and then decreased with the prolongation of culture time. Compared with the high concentration of the HQ treatment condition (3.0 mmol·L^−1^), low concentrations of HQ are more beneficial to the synthesis of arbutin in suspension cells. This may be because high concentrations of HQ and prolonged treatment are detrimental to the accumulation of arbutin in the cells.

### 2.2. Effect of HQ on Metabolite and Gene Changes in V. dunalianum Suspension Cells Based on Metabolomics and Transcriptomics

The content of arbutin in suspension cells treated with 0.5 mmol·L^−1^ HQ for 12 h reached a maximum. To comprehensively elucidate the impact of precursor HQ feeding on the biosynthesis of metabolites and gene expressions, metabolomics and transcriptomics analyses were performed on suspension cells treated with 0 and 0.5 mmol·L^−1^ HQ for 12 h.

#### 2.2.1. Differentially Expressed Metabolites (DEMs) Identified in Suspension Cells Treated with 0 and 0.5 mmol·L^−1^ HQ for 12 h by Metabolomics Analysis

**Figure 2:**
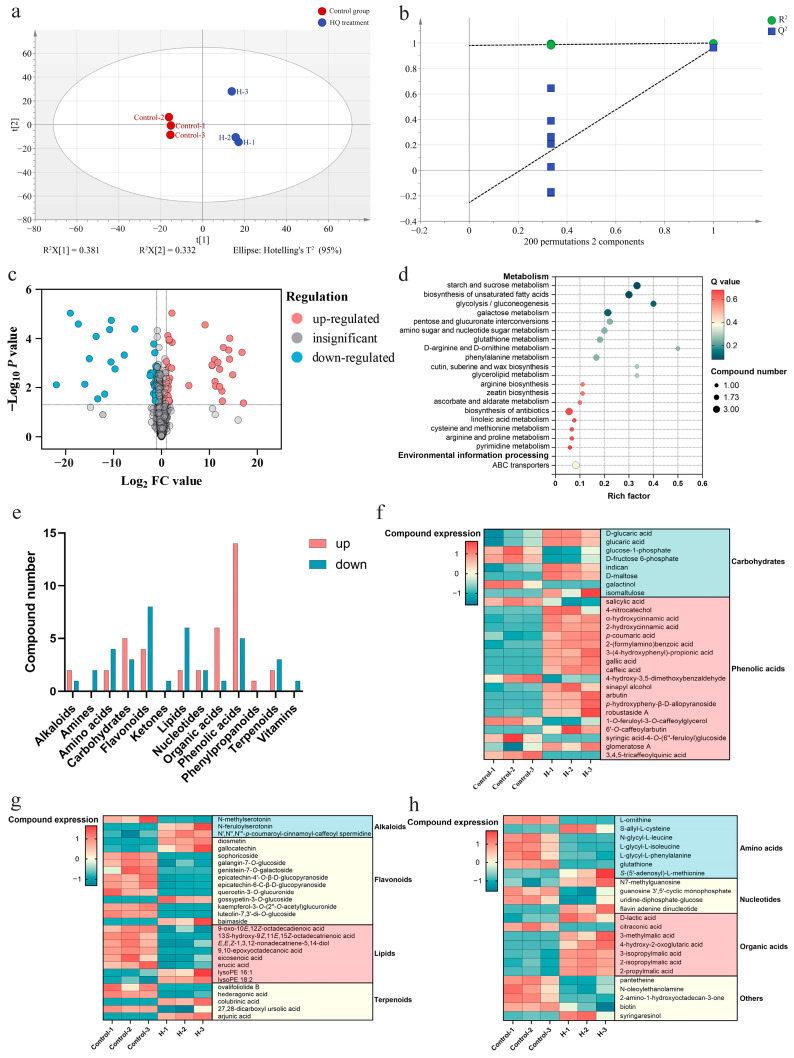
Analysis of DEMs in *V. dunalianum* suspension cells treated with 0 and 0.5 mmol·L^−1^ HQ for 12 h. (**a**) PLS-DA model with the model parameter values of R^2^X (cum) = 0.713, R^2^Y (cum) = 0.999, Q^2^ (cum) = 0.962. (**b**) Permutation test with 200 iterations and the intercepts of R^2^ = 0.981, Q^2^ = −0.252. (**c**) Volcano plot of differential metabolites. (**d**) KEGG enrichment analysis of differential metabolites with top 20 pathways. (**e**) Classification of differential metabolites. (**f**–**h**) Expression of differential metabolites.

Before obtaining the DEMs, the partial least squares-discriminant analysis (PLS-DA) model was employed to distinguish two groups of samples, revealing that there were significant differences between groups (Figure 2a,b). Then a total of 78 DEMs were identified in suspension cells treated with 0 and 0.5 mmol·L^−1^ HQ for 12 h, including 40 up-regulated and 38 down-regulated metabolites in suspension cells treated with HQ compared with the control group (Figure 2c and Table S1). KEGG (Kyoto Encyclopedia of Genes and Genomes) pathway analysis showed that these DEMs were most enriched in starch and sucrose metabolism (ko00500), biosynthesis of unsaturated fatty acids (ko01040), and glycolysis and gluconeogenesis (ko00010) pathways (Figure 2d).

The DEMs primarily consisted of phenolic acids and flavonoids, with a higher number of up-regulated DEMs observed for phenolic acids, carbohydrates, and organic acids (Figure 2e–h). The heatmap of DEMs suggested the addition of HQ greatly improved the accumulation of phenolic acid compounds in suspension cells. It is worth noting that CA was also a significantly up-regulated metabolite in suspension cells treated with HQ (Figure 2f), suggesting that the addition of HQ also promoted CA synthesis in suspension cells.

#### 2.2.2. Differential Expression Genes (DEGs) Identified in Suspension Cells Treated with 0 and 0.5 mmol·L^−1^ HQ for 12 h by Transcriptomics Analysis

Although HPLC results and metabolomics analysis showed that the addition of HQ enhances the synthesis of arbutin and CA, the genes involved in the synthesis of these two compounds in *V. dunalianum* are currently unclear. Therefore, the samples from the control and HQ-treated (treated with 0.5 mmol·L^−1^ HQ for 12 h) groups were further used for transcriptomics analysis. A total of 185,738,256 bp and 201,313,356 bp raw reads were obtained from *V. dunalianum* suspension cells treated with 0 and 0.5 mmol·L^−1^ HQ for 12 h, which were filtered to obtain 185,045,696 bp (99.63%) and 200,667,084 bp (99.68%) clean reads respectively (Table S2). The Q30 percentages of total clean reads in samples ranged from 93.32 to 94.49%, and GC percentages ranged from 46.34 to 47.04% (Table S2), indicating that the transcriptomic data were high-quality and could be used for further differential expression genes (DEGs) analysis. Subsequently, a total of 105,147 unigenes were assembled (Table S3) and annotated by the GO (Gene Ontology) function and KEGG pathway.

**Figure 3:**
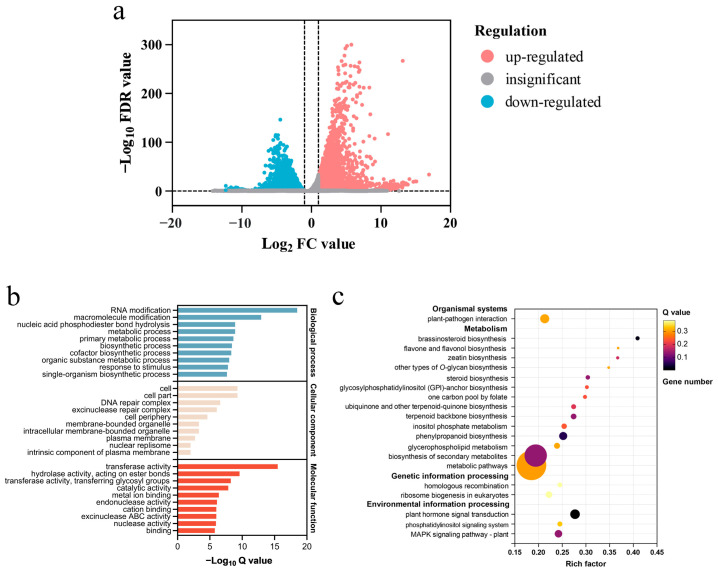
Analysis of DEGs in V. dunalianum suspension cells treated with 0 and 0.5 mmol·L^−1^ HQ for 12 h. (**a**) Volcano plot of DEGs. (**b**) GO enrichment analysis of DEGs. (**c**) KEGG enrichment analysis of DEGs.

Transcriptome sequencing results showed that there were 10,628 DEGs in suspension cells treated with 0 and 0.5 mmol·L^−1^ HQ for 12 h, including 4125 up-regulated and 6503 down-regulated genes in HQ-treated suspension cells compared with the control group (Figure 3a and Table S3). GO analysis revealed that RNA modification, cell, and transferase activity were the most enrichment terms in biological process, cellular component, and molecular function ontologies respectively (Figure 3b). Furthermore, KEGG analysis demonstrated that DEGs were predominantly enriched in pathways related to plant hormone signal transduction (ko04075), brassinosteroid biosynthesis (ko00905), and phenylpropanoid biosynthesis (ko00940) (Figure 3c).

### 2.3. Integrated Metabolomics and Transcriptomics Analyze the Synthetic Pathway of Arbutin in V. dunalianum Suspension Cells Feeding with 0.5 mmol·L^−1^ HQ for 12 h

The transcriptomics and metabolomics analyses were performed on *V. dunalianum* suspension cells treated with 0 and 0.5 mmol·L^−1^ HQ for 12 h. These results were assessed together for a comprehensive understanding of how HQ affects the synthesis of arbutin.

**Figure 4:**
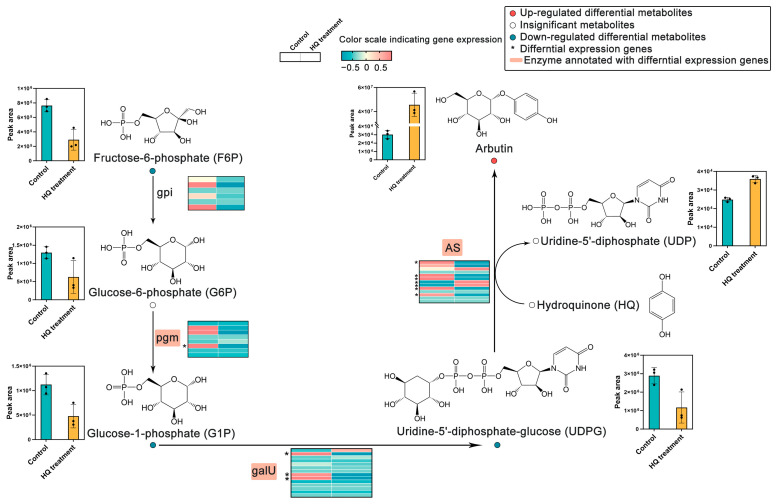
Gene expression and metabolite change in the biosynthetic pathway of arbutin in suspension cells treated with 0.5 mmol·L^−1^ HQ for 12 h.

Arbutin is generated through the glycosylation of HQ, which is an essential precursor in its synthesis [6]. Based on a reported microbial arbutin synthesis pathway [29], a pathway diagram was constructed that included an expression heat map of genes and metabolites involved in arbutin formation (Figure 4). In this synthesis pathway, uridine-diphosphate-5′-glucose (UDPG) acted as a glucosyl donor while HQ functioned as a glucosyl acceptor. Under the catalysis of arbutin synthase (AS, EC 2.4.1.218), UDPG transferred the glucosyl to HQ, thereby generating arbutin and uridine-5′-diphosphate (UDP). UDPG is a metabolite of fructose-6-phosphate (F6P) in the glycolytic pathway. Upstream of this pathway, most genes encoding glucose-6-phosphate isomerase (gpi, EC: 5.3.1.9), phosphoglucomutase (pgm, EC: 5.4.2.2), and glucose-1-phosphate uridylyltransferase (galU, EC:2.7.7.9) exhibited down-regulated expression in suspension cells treated with HQ (Figure 4). As a crucial enzyme for synthesizing arbutin, transcriptomics sequencing data analysis found that two DEGs (Unigene0063512 and Unigene0063513) annotated as *AS* were significantly up-regulated in suspension cells treated with HQ, indicating these two genes may play an important role in the arbutin synthesis (Figure 4).

Metabolites associated with DEGs in the arbutin synthetic pathways also underwent changes. In the synthetic pathway, F6P, glucose-1-phosphate (G1P), and UDGP were identified as down-regulated DEMs in HQ-treated suspension cells, while glucose 6-phosphate (G6P) also decreased although it was not a DEM (Figure 4 and Table 2). All these metabolites are precursors of arbutin synthesis. Arbutin was identified as an up-regulated DEM. There was a significantly higher amount of arbutin and an increased peak area of UDP in suspension cells treated with HQ compared with control (Figure 4 and Table 2). These findings were consistent with the results of the transcriptomics analysis.

### 2.4. Speculation on the Potential Synthesis Pathway of CA in V. dunalianum Suspension Cells Feeding with 0.5 mmol·L^−1^ HQ for 12 h Based on Metabolomics and Transcriptomics

**Figure 5:**
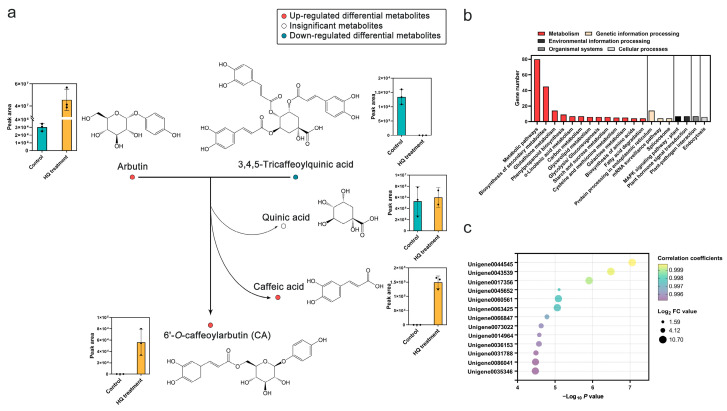
Proposed synthesis pathway for CA and associated genes in suspension cells treated with 0.5 mmol·L^−1^ HQ for 12 h. (**a**) Potential syntheyic route of CA (**b**) Main KEGG pathways of DEGs positively correlated to CA synthesis (correlation coefficients > 0.9 and *p* value < 0.01). (**c**) 13 DEGs correlated significantly with CA synthesis (correlation coefficients > 0.995 and *p* value < 0.01).

In the above metabolomics analysis, the addition of HQ not only facilitated arbutin synthesis but also promoted CA synthesis in suspension cells. However, the synthetic pathway of CA has not been elucidated so far. Based on differential metabolites analysis and previous research [23], this study speculated that caffeoylquinic acid acted as caffeoyl donors and arbutin as a caffeoyl receptor, and the caffeoyl group from caffeoylquinic acids was transferred to the glucose-6′ of arbutin to form CA under the catalysis of caffeoyl transferase, while simultaneously generating caffeic acid and quinic acid (Figure 5a). Three caffeoylquinic acids, namely cryptochlorogenic acid (4-*O*-caffeoylquinic acid), neochlorogenic acid (5-*O*-caffeoylquinic acid), and 3,4,5-tricaffeoylquinic acid, were identified in suspension cells by ultrafiltration-ultra-performance liquid chromatography and quadrupole-time-of-flight mass spectrometry (UF-UPLC-Q/TOF-MS/MS) (Table 2). Among them, 3,4,5-tricaffeoylquinic acid was considered as a potential substrate in the CA synthesis pathway due to it being a down-regulated DEM, exhibiting a lower amount in suspension cells treated with HQ compared with the control (Figure 5a and Table 2). At the same time, there were significantly higher levels of CA, caffeic acid, and a slightly increased peak area of quinic acid in suspension cells treated with HQ (Figure 5a and Table 2). The changes in these metabolites align with the potential synthesis route of CA.

To further analyze the genes related to CA biosynthesis, the correlation analysis between gene expression and CA synthesis was performed. A total of 462 DEGs was positively associated with CA synthesis with Pearson correlation coefficients > 0.9 and *p* value < 0.01 (Table S4). These DEGs were mostly annotated into metabolic pathways, biosynthesis of secondary metabolites, and glutathione metabolism pathways in the KEGG database (Figure 5b). Thirteen DEGs were significantly correlated to CA with correlation coefficients > 0.995 (Figure 5c). Figure 5c shows the correlation coefficients and log_10_-fold change (FC) values of these DEGs. Of these genes, Unigene0044545, Unigene0043539, and Unigene0017356 exhibited higher FC values and possessed higher correlation coefficients correlated to CA, suggesting their potential roles in CA synthesis.

## 3. Discussion

Arbutin and CA are the principal compounds in *V. dunalianum*, which exhibit excellent whitening properties by inhibiting melanin formation [7]. However, the utilization of plants as a source of metabolites is constrained by limited resources and the potential destruction of wild populations. Despite the employment of cell suspension for metabolite production, obtaining sufficient secondary metabolites remains challenging due to the lack of suitable induction conditions. Previous research has reported low levels of arbutin and CA in *V. dunalianum* suspension cells [21]. Additionally, the biosynthesis pathways of arbutin and CA in *V. dunalianum* are not yet fully understood, which greatly restricts the development and application of these two bioactive compounds. In this study, HQ was introduced as a precursor into *V. dunalianum* suspension cells to improve the synthesis of arbutin and CA. The results demonstrated that the addition of HQ efficiently promoted the synthesis of arbutin and CA. A combined analysis of transcriptomics and metabolomics was performed to analyze their biosynthetic mechanism.

### 3.1. HQ Was Essential for Arbutin Biosynthesis in V. dunalianum Suspension Cells

Arbutin is a natural phenolic glycoside compound, which was isolated from the leaves of species belonging to numerous families, such as Ericaceae, Rosaceae, Saxiferaceae, Rubiaceae, and Fabaceae [30]. Its synthesis has received immense attention due to its extensive applications. As the precursor of arbutin synthesis, HQ has been used for the production of arbutin in plant tissue culture [22]. In this study, arbutin content was significantly increased in *V. dunalianum* suspension cells when fed with different concentrations of HQ (0.5, 1.0, and 3.0 mmol·L^−1^) for different treatment times (6, 12, and 48 h). These results demonstrated the ability of *V. dunalianum* suspension cells to transform exogenous HQ into arbutin. The accumulation of arbutin in cells reached its maximum when treated with 0.5 mmol·L^−1^ HQ for 12 h, subsequently declining with further prolongation of the treatment time. This trend may be due to the conversion of arbutin to CA or its release from the cells. Moreover, HQ is considered to be an intermediate product of lignin degradation by microorganisms in forest trees [31]. However, the limited synthesis of arbutin in the *V. dunalianum* control group was attributed to the challenge of synthesizing sufficient HQ. The exogenous addition of HQ provides sufficient precursor for the synthesis of arbutin and induces the expression of enzymes involved in its synthesis, thus promoting arbutin production.

The biosynthetic pathway of arbutin in suspension cells was further analyzed by identifying the DEMs and DEGs by metabolomic and transcriptomic analyses. In the biosynthetic pathway of arbutin, UDPG was identified as the glucosyl donor, which was a DEM. UDPG is formed from a glycolytic pathway, through F6P, G6P, and G1P, respectively catalyzed by gpi, pgm, and galU [32]. In this study, F6P, G6P, G1P, and UDPG exhibited low levels in suspension cells treated with HQ. As they are consumed as precursors for arbutin formation, their levels are down-regulated. Correspondingly, the expressions of related genes annotated into gpi, pgm, and galU were also observed to be down-regulated in suspension cells treated with HQ, which corresponds to inadequate metabolite accumulation. Moreover, AS catalyzed the conversion of UDGP and HQ to arbutin and UDP, resulting in high amounts of arbutin and UDP in HQ-treated suspension cells. As a member of the glycosyltransferase family, AS is a crucial enzyme for the synthesis of arbutin [33,34]. In this study, Unigene0063512 and Unigene0063513 were identified as two up-regulated DEGs annotated into AS, suggesting their vital role in arbutin synthesis.

### 3.2. The Potential Synthetic Route of CA in V. dunalianum Suspension Cells

The highest content of CA was found in the leaf buds of *V. dunalianum*, despite it was also reported in other species [10,35]. Hence, the high accumulation of CA in *V. dunalianum* attracted the attention of researchers. Ding et al. performed de novo transcriptome sequencing of the leaf buds of *V. dunalianum* to investigate the potential pathways of CA [23]. Based on the catalytic mechanism of acyltransferase reported in the literature, the authors speculated that arbutin *O*-caffeoyltransferase, chlorogenic acid: glucaric acid *O*-caffeoyl transferase (CGT), and anthocyanin *O*-hydroxycinnamoyl transferase play an important role in the three putative pathways of CA synthesis. These enzymes respectively catalyze the transfer of the acyl group from caffeoyl-CoA, chlorogenic acid, and 1-*O*-hydroxycinnamoyl-β-D-glucose to glucose-6′ in arbutin, resulting in the formation of CA. The results of this study indicated that CA synthesis in *V. dunalianum* is more likely to involve the transfer of the acyl group of caffeoylquinic acid to glucose-6′ in arbutin to form CA, quinic acid, and caffeic acid catalyzed by caffeoyl transferase. As an acyltransferase, CGT could catalyze the formation of caffeoylglucarate using chlorogenic acid (5-*O*-caffeoylquinate) as an acyl donor [36,37]. Although chlorogenic acid was not detected in suspension cells, there were three derivatives of caffeoylquinic acid in cells. Among these, 3,4,5-tricaffeoylquinic acid was a down-regulated DEM, which could potentially be a substrate for CA synthesis. In addition, the peak areas of caffeic acid and quinic acid were higher in cells treated with HQ compared to the control group, further supporting this biosynthetic route. Unfortunately, the genes cannot be annotated into CGT in the KEGG database due to limited information about CGT. Nevertheless, Pearson correlation analysis showed that genes Unigene0044545, Unigene0043539, and Unigene0017356 exhibited higher FC values and possessed higher correlation coefficients with CA, suggesting their importance in CA synthesis. Unfortunately, these genes lacked detailed annotations in current databases for further analysis.

The present findings served as a valuable reference for future endeavors to identify key regulators involved in CA synthesis. However, this study relies on transcriptomics and metabolomics analyses and falls short of providing direct evidence for enzyme activities. Experimental enzyme assays are necessary to substantiate the roles of the proposed enzymes, particularly CGT. In addition, to gain a comprehensive understanding of CA biosynthesis in *V. dunalianum*, future research should encompass a wider array of genes and enzymes.

## 4. Materials and Methods

### 4.1. Plant Material and Cell Suspension Culture Establishment

The original material of *V. dunalianum* was collected from Wuding County, Yunnan Province, China, in April 2015. The collected leaves of *V. dunalianum* were sterilized immediately and used to induce callus. Cell suspension cultures were established using callus cells, which were induced from the leaves of *V. dunalianum* [20]. Specifically, 2.64 g healthy and friable callus was selected and inoculated into a 150 mL conical flask containing 50 mL improved liquid woody plant medium with 40 g·L^−1^ sucrose, 1.5 mg·mL^−1^ zeatin, and 0.15 mg·mL^−1^ naphthylacetic acid. The suspension cells were maintained on a rotary shaker cultured at 149 rpm and 25 °C in the dark. Subculturing of the suspension cells was performed every seven days for subsequent experiments.

### 4.2. Precursor Feeding Experiment

The precursor HQ (99% purity, Aladdin Industrial Corporation, Shanghai, China) was added to the cell suspension culture at an exponential growth period on the 9th day of cultivation under sterile conditions [20]. The final concentrations of added HQ in culture media were 0.5, 1.0, and 3.0 mmol·L^−1^, respectively. No HQ was added as a control. All groups were cultured on a shaker with a rotary shaker of 149 rpm and 25 °C under dark conditions. The samples were harvested at 6 h, 12 h, and 48 h. Each group contained three biological replicates. All samples were washed three times with sterilized water and immediately frozen in liquid nitrogen for subsequent analysis.

### 4.3. Arbutin Extraction and HPLC Analysis

The dried powders (0.5 g) of each sample were extracted with 10 mL methanol in an ultrasound-assisted water bath. These extracts were filtered by a nylon syringe filter (SCAA-104, ANPEL, Shanghai, China) of 0.22 μm. The arbutin concentration of all samples was determined by Agilent 1260 HPLC system (Agilent Technologies, Santa Clara, CA, USA) using CAPCELL PAK MGII C18 column (4.6 × 250 mm, 5 μm; Shiseido Co., Ltd., Tokyo, Japan) and a detected wavelength at 210 nm. The mobile phase consisted of acetonitrile (A) and water (B) with a flow rate of 0.8 mL·min^−1^. The elution procedure employed was as follows: 0–5 min, 7 to 17% A; 5–20 min, 17% A; 20–21 min, 17 to 57% A; 21–31 min, 57% A; 31–32 min, 57 to 95% A; 32–42 min, 95% A; 42–45 min, 95 to 5% A.

The content of arbutin in cells was calculated by the following formula:

Arbutin content (mg·g^−1^) = (*C* × *V*)/*M* (1)

where *C*, *V*, and *M* are the concentration of arbutin (mg·mL^−1^), extract volume (mL), and sample weight (g), respectively.

### 4.4. Metabolomics Analysis

Suspension cells treated with 0 and 0.5 mmol·L^−1^ HQ for 12 h were selected for metabolomics analysis. The freeze-dried samples were crushed using a mixer mill (MM 400, Retsch, Haan, Germany) with a zirconia bead for 1.5 min at 30 Hz. The 0.1 g dry powder from each sample was extracted at 4 °C with 1.0 mL 70% aqueous methanol overnight, followed by centrifugation at 10,000× *g* for 10 min. The extracts were filtrated by a filter of 0.22 μm for UF-UPLC-Q/TOF-MS/MS analysis.

The UF-UPLC-Q/TOF-MS/MS analysis was performed on Shim-pack UFLC SHIMADZU CBM30A system (Shimadzu, Kyoto, Japan) coupled with Applied Biosystems 6500 QTRAP (Applied Biosystems, Foster City, CA, USA) with Waters ACQUITY UPLC HSS T3 C18 column (2.1 × 100 mm, 1.8 μm; Waters, Milford, MA, USA) with an injection volume of 2 μL. A gradient elution procedure comprising acetonitrile (A) and water with 0.04% acetic acid (B) was developed with a flow rate of 0.4 mL·min^−1^. The flow elution procedure employed was as follows: 0–11 min, 5 to 95% A; 11–12 min, 95% A; 12–12.1 min, 95 to 5% A; 12.1–15 min, 5% A. The mass spectrometry was recorded in both positive and negative ion mode, with a source temperature of 500 °C, ion spray voltage of 5500 V, and scan range of 500–1000 *m*/*z*.

The raw data files obtained from UF-UPLC-Q/TOF-MS/MS analysis were filtering, peak detection, alignment, and calculations were performed using Analyst 1.6.1 software. To generate a matrix containing fewer biased and redundant data, peaks were checked manually for signal/noise ratio > 10 and in-house software written in Perl was used to remove the redundant signals caused by different isotopes, in-source fragmentation, K^+^, Na^+^, and NH_4_^+^ adduct, and dimerization. The characteristics of metabolites were obtained from simple screening of data with their retention time, mass-to-charge ratio and peak alignment, molecular weight of the compound, and mass deviation and adduct ion information. The metabolites were identified by searching the internal database, public databases of MassBank (https://massbank.eu/, accessed on 12 April 2022), KNApSAcK (https://ngdc.cncb.ac.cn/databasecommons/, accessed on 12 April 2022), HMDB (https://hmdb.ca/, accessed on 12 April 2022), and METLIN (https://metlin.scripps.edu, accessed on 12 April 2022), comparing the *m*/*z* values, the retention time, and the fragmentation patterns with the standards [38,39]. A normalization method for peak area was employed and the area of each peak represented the relative content of the corresponding metabolite. Based on metabolite data, PLS-DA was conducted to analyze the difference in metabolites between samples using SIMCA 14.1. The significant difference thresholds of standards of variable importance in projection (VIP) value > 1, *p* value < 0.05 in the *t*-test, and FC value > 2 or < 0.5 were used to identify the DEMs. Then, DEMs were mapped to the KEGG database for function annotation.

### 4.5. Transcriptomics Analysis

Suspension cells treated with 0 and 0.5 mmol·L^−1^ HQ for 12 h were also used for transcriptomics analysis. Total RNAs of samples were extracted using a Trizol reagent kit (Invitrogen, Carlsbad, CA, USA) according to the manufacturer’s protocol. The purity and concentration of the RNA were assessed using NanoDrop spectrophotometry (Thermo Fisher Scientific, Waltham, MA, USA) and the Agilent 2100 Bioanalyzer (Agilent Technologies, CA, USA), respectively. High-quality RNA samples were selected for the preparation of the cDNA library. The constructed library was sequenced using an Illumina novaseq 6000 sequencer (Gene Denovo Biotechnology Co., Guangzhou, China).

The high-quality clean reads were obtained from raw reads filtered by fastp and then were assembled using Trinity to obtain unigenes. The expression of unigenes was calculated and normalized to RPKM (Reads Per Kb per Million reads). DEGs between samples were identified using RPKM data based on false discovery rate (FDR) value and FC value, applying thresholds of FDR value < 0.05 and |log_2_ FC value| > 1. The function and pathway of DEGs were annotated using Nr (Non-redundant protein sequence database), SwissProt, KEGG, and GO databases.

### 4.6. Transcriptomics and Metabolomics Correlation Analysis

Differences in gene expression and metabolite levels were obtained from transcriptomics and metabolomics. However, transcription and metabolism do not occur independently in biological systems. Correlation analysis was used to reveal the regulatory mechanism between gene expression and metabolites. To obtain the association characteristics, the correlation analysis was conducted based on the two sets of data on gene expression and metabolite abundance. Pearson correlation coefficients were performed to further analyze underlying genes for CA biosynthesis by calculating the correlation coefficients of expression of DEGs with CA. DEGs that were positively associated with CA (correlation coefficients > 0.9 and *p* value < 0.01) were considered potential genes related to CA synthesis. Specifically, DEGs that exhibited correlation coefficients > 0.995 (*p* value < 0.01) were correlated significantly with CA synthesis.

### 4.7. Statistical Analysis

The experimental data were processed statistically using Microsoft Excel Significant differences among samples were determined using one-way analysis of variance (ANOVA) and Duncan’s multiple range test on SPSS 23.0. All the experimental data were presented as the mean ± standard deviation with three replicates, and visualized using GraphPad Prism 9.

## 5. Conclusions

In this study, precursor feeding was selected as a strategy to enhance the production of arbutin and CA in suspended cells of *V. dunalianum*. The results of HPLC and UF-UPLC-Q/TOF-MS/MS analyses suggested that the addition of precursor HQ promoted the synthesis of arbutin and CA. Besides, the molecular mechanism of arbutin and CA biosynthesis in suspension cells of *V. dunalianum* under HQ treatment was revealed through the identification of DEGs and DEMs using metabolomics and transcriptomics. The results of the conjoint analysis indicated that arbutin was formed from UDPG and HQ catalyzed by AS, and two up-regulated *AS* genes play key roles in arbutin biosynthesis. In addition, the potential synthetic route of CA in suspended cells of *V. dunalianum* was arbutin and 3,4,5-tricaffeoylquinic acid catalyzed by CGT to form CA, caffeic acid, and quinic acid. Overall, this study indicated that precursor feeding of HQ in suspension cells of *V. dunalianum* was a promising strategy for mass biosynthesis for arbutin and CA. These findings provide new information and insights for future research on CA biosynthesis in *V. dunalianum*.

## Figures and Tables

**Table 1:** The content of arbutin in control and HQ-treated suspension cells of *V. dunalianum*.

Concentration of Hydroquinone (mmol·L^−1^)	Arbutin Content (mg·g^−1^)		
	6 h	12 h	48 h
0.5	5.96 ± 0.08 ***	15.52 ± 0.28 ***	0.59 ± 0.00 ****
1.0	6.81 ± 0.01 ****	9.58 ± 0.38 ***	3.30 ± 0.15 **
3.0	3.21 ± 0.04 ***	4.49 ± 0.10 ***	0.25 ± 0.01 ***
Control	0.00	0.00	0.00

The data represented mean ± SE of three replicates (n = 3). Asterisk symbols (*) indicate significant differences according to Duncan’s multiple range test (** *p* < 0.01; *** *p* < 0.001; **** *p* < 0.0001).

**Table 2:** The metabolites related to the biosynthesis of arbutin and CA in suspension cells treated with 0.5 mmol·L^−1^ HQ for 12 h.

Compounds	Log_2_ FC Value (Control vs. HQ Treatment)	*p* Value	Regulation
3,4,5-tricaffeoylquinic acid	−10.54	0.0009	down
arbutin	10.56	0.0013	up
6′-*O*-caffeoylarbutin (CA)	12.61	0.0139	up
caffeic acid	14.02	0.0003	up
cryptochlorogenic acid	−0.18	0.6233	insignificant
fructose-6-phosphate (F6P)	−1.39	0.0001	down
glucose-6-phosphate (G6P)	−1.05	0.9163	insignificant
glucose-1-phosphate (G1P)	−1.23	0.0001	down
neochlorogenic acid	−0.12	0.8723	insignificant
quinic acid	0.47	0.3995	insignificant
uridine-5′-diphosphate (UDP)	0.53	0.0011	insignificant
uridine-diphosphate-5′-glucose (UDPG)	−1.31	0.0351	down

## Data Availability

The data are contained within the article.

## Supplementary Materials

The following supporting information can be downloaded at: https://www.mdpi.com/article/10.3390/ijms25147760/s1.

## References

[1] Nahar L., Al-Groshi A., Kumar A., Sarker S.D. (2022). Arbutin: Occurrence in plants, and its potential as an anticancer agent. Molecules.

[2] Agarwal N., Rai A.K., Singh S.P. (2021). Biotransformation of hydroquinone into α-arbutin by transglucosylation activity of a netagenomic amylosucrase. 3 Biotech.

[3] Jin J.L., Liu Y., Jiang C., Shen Y.F., Chu G.Y., Liu C., Jiang L.J., Huang G.R., Qin Y.F., Zhang Y.J. (2022). Arbutin-modified microspheres prevent osteoarthritis progression by mobilizing local anti-inflammatory and antioxidant responses. Mater. Today Bio..

[4] Jurica K., Gobin I., Kremer D., Čepo D.V., Grubešić R.J., Karačonji I.B., Kosalec I. (2017). Arbutin and its metabolite hydroquinone as the main factors in the antimicrobial effect of strawberry tree (*Arbutus unedo* L.) leaves. J. Herb. Med..

[5] Su Y.B., Sun X.W., Wu R.X., Zhang X., Tu Y.Z. (2020). Molecular spectroscopic behaviors of beta-arbutin in anti-skin cancer. Spectrosc. Lett..

[6] Shen X.L., Wang J., Wang J., Chen Z.Y., Yuan Q.P., Yan Y.J. (2017). High-level de novo biosynthesis of arbutin in engineered *Escherichia coli*. Metab. Eng..

[7] Xu M., Lao Q.C., Zhao P., Zhu X.Y., Zhu H.T., Luo X.L., Yang C.R., He J.H., Li C.Q., Zhang Y.J. (2014). 6′-*O*-Caffeoylarbutin inhibits melanogenesis in zebrafish. Nat. Prod. Res..

[8] Adem S., Eyupoglu V., Sarfraz I., Rasul A., Zahoor A.F., Ali M., Abdalla M., Ibrahim I.M., Elfiky A.A. (2021). Caffeic acid derivatives (CAFDs) as inhibitors of SARS-CoV-2: CAFDs-based functional foods as a potential alternative approach to combat COVID-19. Phytomedicine.

[9] Wang Y.P., Wang Y.D., Liu Y.P., Cao J.X., Yang M.L., Wang Y.F., Khan A., Zhao T.R., Cheng G.G. (2022). 6′-*O*-Caffeoylarbutin from Que Zui tea ameliorates acetaminophen-induced liver injury via enhancing antioxidant ability and regulating the PI3K signaling pathway. Food Funct..

[10] Zhao P., Tanaka T., Hirabayashi K., Zhang Y.J., Yang C.R., Kouno I. (2008). Caffeoyl arbutin and related compounds from the buds of *Vaccinium dunalianum*. Phytochemistry.

[11] Li N., Zeng W.L., Luo X.L., Yang C.R., Zhang Y.J., Ding Y., Zhao P. (2018). A new arbutin derivative from the leaves of *Vaccinium dunalianum* Wight. Nat. Prod. Res..

[12] Arya S.S., Rookes J.E., Cahill D.M., Lenka S.K. (2020). Next-generation metabolic engineering approaches towards development of plant cell suspension cultures as specialized metabolite producing biofactories. Biotechnol. Adv..

[13] Malik S., Cusidó R.M., Mirjalili M.H., Moyano E., Palazón J., Bonfill M. (2011). Production of the anticancer drug taxol in *Taxus baccata* suspension cultures: A review. Process Biochem..

[14] Yu Y., Wang T., Wu Y.C., Zhou Y.H., Jiang Y.Y., Zhang L. (2019). Effect of elicitors on the metabolites in the suspension cell culture of *Salvia miltiorrhiza* Bunge. Physiol. Mol. Biol. Plants.

[15] Du L.D., Li D.M., Zhang J.J., Du J., Luo Q.Z., Xiong J.H. (2020). Elicitation of *Lonicera japonica* Thunb suspension cell for enhancement of secondary metabolites and antioxidant activity. Ind. Crop. Prod..

[16] Rajan M., Feba K.S., Chandran V., Shahena S., Mathew L. (2020). Enhancement of rhamnetin production in *Vernonia anthelmintica* (L.) Willd. cell suspension cultures by eliciting with methyl jasmonate and salicylic acid. Physiol. Mol. Biol. Plants.

[17] Luo J., He G.Y. (2004). Optimization of elicitors and precursors for paclitaxel production in cell suspension culture of *Taxus chinensis* in the presence of nutrient feeding. Process Biochem..

[18] John R., Shajitha P.P., Devassy A., Mathew L. (2018). Effect of elicitation and precursor feeding on accumulation of 20-hydroxyecdysone in *Achyranthes aspera* Linn. cell suspension cultures. Physiol. Mol. Biol. Plants.

[19] Rattan S., Kumar A., Kumar D., Warghat A.R. (2022). Enhanced production of phenylethanoids mediated through synergistic approach of precursor feeding and light regime in cell suspension culture of *Rhodiola imbricata* (Edgew.). Appl. Biochem. Biotechnol..

[20] Li C.R., Fu L., Liu Y., Yang X.Q., Zhu G.L., Xie S.D., Ma H.C., Zhao P. (2022). Optimization of cell suspension culture conditions of *Vaccinium dunalianum*. Chin. Bull. Bot..

[21] Wu B.X., Fu L., Li C.R., Liu Y., Tang J.R., Yang X.Q., Ma H.C., Zhao P. (2022). LC-MS analysis of secondary metabolites in suspension culture cells of *Vaccinium dunalianum*. J. Southwest For. Univ. (Nat. Sci.).

[22] Xu K.X., Xue M.G., Li Z., Ye B.C., Zhang B. (2022). Recent progress on feasible strategies for arbutin production. Front. Bioeng. Biotechnol..

[23] Ding Y., Xiong H., Li N., Song J., Zheng Y.L., Liu X.Z., Zhao P. (2017). De novo transcriptome sequencing of *Vaccinium dunalianum* Wight to investigate arbutin and 6′-*O*-caffeoylarbutin synthesis. Russ. J. Plant Physiol..

[24] Zhang S.M., Sun F.L., Zhang C.Q., Zhang M.T., Wang W.W., Zhang C., Xi Y.J. (2022). Anthocyanin biosynthesis and a regulatory network of different-colored wheat grains revealed by multiomics analysis. J. Agric. Food Chem..

[25] Li Y.P., Xie Z.Y., Huang Y., Zeng J.Y., Yang C., Yuan L., Wang Y., Li Y.Q. (2024). Integrated metabolomic and transcriptomic analysis provides insights into the flavonoid formation in different *Glycyrrhiza* species. Ind. Crop. Prod..

[26] Lv Y.Y., Zhu J.J., Huang S.H., Xing X.L., Zhou S., Yao H., Yang Z., Liu L., Huang S.S., Miao Y.Y. (2024). Metabolome profiling and transcriptome analysis filling the early crucial missing steps of piperine biosynthesis in *Piper nigrum* L.. Plant J..

[27] Brychkova G., de Oliveira C.L., Gomes L.A.A., de Souza Gomes M., Fort A., Esteves-Ferreira A.A., Sulpice R., McKeown P.C., Spillane C. (2023). Regulation of carotenoid biosynthesis and degradation in Lettuce (*Lactuca sativa* L.) from seedlings to harvest. Int. J. Mol. Sci..

[28] Chen W.J., Yan J.W., Zheng S., Suo J.W., Lou H.Q., Song L.L., Wu J.S. (2023). Integrated metabolomics, transcriptome and functional analysis reveal key genes are involved in tree age-induced amino acid accumulation in *Torreya grandis* nuts. Int. J. Mol. Sci..

[29] An N., Xie C., Zhou S.B., Wang J., Sun X.X., Yan Y.J., Shen X.L., Yuan Q.P. (2023). Establishing a growth-coupled mechanism for high-yield production of β-arbutin from glycerol in *Escherichia coli*. Bioresour. Technol..

[30] Nycz J.E., Malecki G., Morag M., Nowak G., Ponikiewski L., Kusz J., Switlicka A. (2010). Arbutin: Isolation, X-ray structure and computional studies. J. Mol. Struct..

[31] Yang Y.L., Zhou H., Du G., Feng K.N., Feng T., Fu X.L., Liu J.K., Zeng Y. (2016). A monooxygenase from *Boreostereum vibrans* catalyzes oxidative decarboxylation in a divergent vibralactone biosynthesis pathway. Angew. Chem. Int. Ed. Engl..

[32] Shang Y.Z., Wei W.P., Zhang P., Ye B.C. (2020). Engineering *Yarrowia lipolytica* for enhanced production of arbutin. J. Agric. Food Chem..

[33] Arend J., Warzecha H., Stöckigt J. (2000). Hydroquinone: *O*-glucosyltransferase from cultivated *Rauvolfia* cells: Enrichment and partial amino acid sequences. Phytochemistry.

[34] Hefner T., Arend J., Warzecha H., Siems K., Stöckigt J. (2002). Arbutin synthase, a novel member of the NRD1β glycosyltransferase family, is a unique multifunctional enzyme converting various natural products and xenobiotics. Bioorg. Med. Chem..

[35] Manju M., Varma R.S., Parthasarathy M.R. (1977). New arbutin derivatives from leaves of *Gervillea robusta* and *Hakea saligna*. Phytochemistry.

[36] Strack D., Gross W. (1990). Properties and activity changes of chlorogenic acid: Glucaric acid caffeoyltransferase from tomato (*Lycopersicon esculentum*). Plant Physiol..

[37] Teutschbein J., Gross W., Nimtz M., Milkowski C., Hause B., Strack D. (2010). Identification and localization of a lipase-like acyltransferase in phenylpropanoid metabolism of tomato (*Solanum lycopersicum*). J. Biol. Chem..

[38] Wishart D.S., Jewison T., Guo A.C., Wilson M., Knox C., Liu Y.F., Djoumbou Y., Mandal R., Aziat F., Dong E. (2013). HMDB 3.0-the human metabolome database in 2013. Nucleic Acids Res..

[39] Zhu Z.J., Schultz A.W., Wang J.H., Johnson C.H., Yannone S.M., Patti G.J., Siuzdak G. (2013). Liquid chromatography quadrupole time-of-flight mass spectrometry characterization of metabolites guided by the METLIN database. Nat. Protoc..

